# Identifying pathogenic variants in rare pediatric neurological diseases using exome sequencing

**DOI:** 10.1038/s41598-024-75020-0

**Published:** 2024-10-21

**Authors:** Kazuyuki Komatsu, Mitsuhiro Kato, Kazuo Kubota, Shinobu Fukumura, Keitaro Yamada, Ikumi Hori, Kenji Shimizu, Sachiko Miyamoto, Kaori Yamoto, Takuya Hiraide, Kazuki Watanabe, Shintaro Aoki, Shogo Furukawa, Taiju Hayashi, Masaharu Isogai, Takuma Harasaki, Mitsuko Nakashima, Hirotomo Saitsu

**Affiliations:** 1 Department of Biochemistry, Hamamatsu University School of Medicine, Hamamatsu, 431- 3192 Japan; 2 Department of Pediatrics, Showa University School of Medicine, Tokyo, 142-8555 Japan; 3 Department of Pediatrics, Gifu University Graduate School of Medicine, Gifu, 501-1194 Japan; 4 Division of Clinical Genetics, Gifu University Hospital, Gifu, 501-1194 Japan; 5 Department of Pediatrics, Sapporo Medical University School of Medicine, Sapporo, 060-8556 Japan; 6 Department of Pediatric Neurology, Central Hospital, Aichi Developmental Disability Center, Kasugai, 486-0392 Japan; 7 Department of Pediatrics and Neonatology, Nagoya City University Graduate School of Medical Sciences, Nagoya, 467-8601 Japan; 8 Department of Pediatrics, Aichi Prefectural Welfare Federation of Agricultural Cooperatives Kainan Hospital, Yatomi, 498-8502 Japan; 9 Division of Medical Genetics, Shizuoka Children’s Hospital, Shizuoka, 420-8660 Japan; 10 Department of Pediatrics, Hamamatsu University School of Medicine, Hamamatsu, 431-3192 Japan; 11 Department of Neurology, Hamamatsu University School of Medicine, Hamamatsu, 431-3192 Japan

**Keywords:** Neurological rare diseases, Exome sequencing, Annotation, *De novo* variant, Splicing abnormality, Genetics, Diseases, Neurology

## Abstract

Variant annotations are crucial for efficient identification of pathogenic variants. In this study, we retrospectively analyzed the utility of four annotation tools (allele frequency, ClinVar, SpliceAI, and Phenomatcher) in identifying 271 pathogenic single nucleotide and small insertion/deletion variants (SNVs/small indels). Although variant filtering based on allele frequency is essential for narrowing down on candidate variants, we found that 13 *de novo* pathogenic variants in autosomal dominant or X-linked dominant genes are registered in gnomADv4.0 or 54KJPN, with an allele frequency of less than 0.001%, suggesting that very rare variants in large cohort data can be pathogenic *de novo* variants. Notably, 38.4% candidate SNVs/small indels are registered in the ClinVar database as pathogenic or likely pathogenic, which highlights the significance of this database. SpliceAI can detect candidate variants affecting RNA splicing, leading to the identification of four variants located 11 to 50 bp away from the exon–intron boundary. Prioritization of candidate genes by proband phenotype using the PhenoMatcher module revealed that approximately 95% of the candidate genes had a maximum PhenoMatch score ≥ 0.6, suggesting the utility of phenotype-based variant prioritization. Our results suggest that a combination of multiple annotation tools and appropriate evaluation can improve the diagnosis of rare diseases.

**Supplementary Information:**

The online version contains supplementary material available at 10.1038/s41598-024-75020-0.

## Introduction

Comprehensive genetic analysis using next-generation sequencing has dramatically improved the diagnostic yield of genetic diseases. Approximately 50% of rare neurodevelopmental diseases have been diagnosed using various next-generation sequencing technologies, including exome or genome sequencing and transcriptome sequencing^1^. The most commonly used method is exome sequencing, which targets the exons of all genes. Human coding exons contain approximately 17,000 single nucleotide variants (SNVs) and small insertion/deletions (Indels)^2^. Exome sequencing can also detect variants in adjacent introns^3^. The SpliceAI score^4^ is used as computational evidence in decision tree for intronic variants using the American College of Medical Genetics/Association of Molecular Pathology (ACMG/AMP) framework^5^. However, there is no consensus on how many bases in an intron should be analyzed. Exome sequencing can also be used to detect copy number variations (CNVs) and, thereby, contributes to genetic diagnosis^1^.

The first step in narrowing down on candidate variants in rare genetic diseases is to exclude common variants. A globally used database for excluding common variants is gnomAD^6^, the largest public open-access reference dataset for human genome allele frequencies (https://gnomad.broadinstitute.org/). It comprises 730,947 exomes and 76,215 genomes in its version 4.0, containing SNVs and indels less than 50 bp in length from all ethnicities. In Japan, the 54KJPN database, which comprises 54,302 genome sequencing data from Japanese individuals, has been curated (https://jmorp.megabank.tohoku.ac.jp/)^7^. Data from individuals affected by severe pediatric diseases and their first-degree relatives were excluded from gnomAD. However, pathogenic heterozygous variants for dominant severe pediatric diseases might still be present because of some factors, such as incomplete penetrance, imprinting, or mosaicism^1^. In practice, rare variants with minor allele frequency equal to or less than 1% are commonly analyzed^1^.

ClinVar is a freely accessible data archive provided by NCBI that offers information on the pathogenic significance and phenotypes of human genome variants^8^. It includes details on the submitter of the variant, classifications of the pathogenic significance of the variants, and other clinical data. Variants submitted to ClinVar are classified as pathogenic (P), likely pathogenic (LP), uncertain significance (VUS), conflicting classifications of pathogenicity, and under other categories. As of July 30, 2024, 369,269 P or LP variants among 2,983,625 total variants are registered (https://clinvarminer.genetics.utah.edu/variants-by-significance). The information in ClinVar is useful for identifying pathogenic variants in the exome; however, the extent to which ClinVar can contribute to diagnostic yield remains to be determined.

In this study, we retrospectively analyzed the utility of four annotation tools (allele frequency, ClinVar, SpliceAI, and Phenomatcher) in identifying pathogenic variants using exome sequencing data from probands with rare neurological diseases. Our findings should contribute to improving the diagnostic yield in exome sequencing analyses.

## Materials and methods

### Probands and initial exome analysis

Experimental protocols were approved by the Institutional Review Board Committee at Hamamatsu University School of Medicine (15–282, 17–163, and 20–207) and Showa University School of Medicine (G219-N and G220-N). Clinical information and peripheral blood samples were obtained after written informed consent was provided from all individuals and/or their legal guardians in agreement with the requirements of Japanese regulations. Using exome sequencing, we analyzed 463 probands with pediatric neurological diseases who were registered in our cohort between April 2016 and March 2024. Their siblings and parents were not included in the 463 probands. Trio-exome analysis was performed for 44 of the probands, including exome sequencing of their parents. The remaining 419 probands were analyzed using proband-only exome analysis. These periods varied for the exome capture and sequencing platforms: SureSelect Human All Exon V6 Kit (Agilent Technologies, Santa Clara, CA) and NextSeq500 (Illumina, San Diego, CA) paired-end sequencing (165 probands); xGen Exome Research Panel kit (IDT, Coralville IA) capture and NextSeq500 sequencing (174 probands) or DNBseq sequencing (33 probands); and Twist Exome 2.0 capture and NovaSeq6000 sequencing (91 probands). Some of these probands have been reported previously^9–17^. Data processing was performed as described previously^18^. To explore the existence of CNVs, we used two CNV detection tools, exome hidden Markovmodel (XHMM)^19^ and jNord methods^20^. The phenotypes of the probands were extracted based on information provided by the attending physicians. Based on the information, we classified the probands into groups with the most pronounced phenotype (Table 1).

**Table 1:** Clinical features and disease inheritance.

Proband’s phenotype	Number of probands	
	Number of probands	With pathogenic variants (%)
Brain malformation	263	144 (54.8)
Seizure	58	44 (75.9)
Abnormal myelination	42	31 (73.8)
Neurodevelopmental delay	39	20 (51.3)
Involuntary movements	15	9 (60.0)
Ataxia	6	6 (100)
Neuromuscular disease	9	6 (66.7)
Spastic paraplegia	8	5 (62.5)
Metabolic	4	2 (50.0)
Others	19	3 (15.8)
Total	463	270
Disease inheritance		
Autosomal dominant	167 (59.6)	
Autosomal recessive	40 (14.3)	
X-linked dominant	22 (7.9)	
X-linked recessive	9 (3.2)	
X-linked (dominant or recessive)	2 (0.7)	
Chromosomal micro deletion/duplication	28 (10.0)	
Not Confirmed	12 (4.3)	
Total	280	

*10 probands had dual phenotypes causing multiple pathogenic variants.

### Retrospective reanalysis of 242 probands possessing pathogenic SNVs/small indels

To evaluate the utility of four annotation tools (allele frequency, ClinVar, SpliceAI, and Phenomatcher) for identifying pathogenic variants, we retrospectively analyzed 242 exome datasets, excluding CNV analysis, as shown in Supplementary Figure S1. Sequenced reads were aligned to the reference genome (GRCh38) and deduplicated using the fq2bam software from Clara Parabricks v4.2.0 (NVIDIA, Santa Clara, CA). After generation of the base quality score recalibration report using the bqsr software, raw variants were called using the haplotypecaller (both from Parabricks v4.2.0, compatible with the Genome Analysis Toolkit version 4.3.0). The generated gVCF file for each proband was combined and quality-filtered using GLNexus (https://github.com/dnanexus-rnd/GLnexus). After removing the common variants in this cohort (Allele Frequency > 0.3) using BCFtools^21^, variants in exons and introns within 50 bp of the exon–intron boundary were annotated with ANNOVAR^22^, using the following databases: gnomADv4.0 exome (730,947 exomes) and 54KJPN for allele frequency, and ClinVar (https://www.ncbi.nlm.nih.gov/clinvar/, version 2024-02-06). We added ClinVar annotation concerning allele ID (ALLELEID), preferred disease name (CLNDN), tag-value pairs of disease database name and identifier (CLNDISDB), review status for the variation ID (CLNREVSTAT), and clinical significance for this single variant (CLNSIG). These variants were also annotated with SpliceAI^4^. Additionally, we ranked the candidate genes with scores based on the Human Phenotype Ontology terms using the PhenoMatcher module (https://github.com/liu-lab/exome_reanalysis)^23^. The most informative common ancestor matrix used for this analysis, created in March 2024 using three datasets (hp.obo, phenotype.hpoa, and genes_to_disease.txt; version 2024-02-08), was downloaded from the human phenotypic ontology webpage (https://hpo.jax.org/). Finally, we also annotated the phenotype information extracted from genemap2.txt, which can be downloaded from Online Mendelian Inheritance in the Man web site (https://www.omim.org/). This information helps in easily checking the names of diseases caused by the genes and their inheritance patterns.

### Evaluation of variant pathogenicity

The definition of pathogenic variant was “Pathogenic” or “Likely pathogenic” according to the ACMG/AMP 2015 guideline^24^ and previously reported pathogenic variants. We confirmed that the phenotypes of the probands were consistent with those mentioned in previous reports by utilizing phenotype information from OMIM and CLNDN. All pathogenic SNVs and CNVs were confirmed using Sanger sequencing, performed on an ABI 3130xl Genetic Analyzer (Applied Biosystems, Foster City, CA), and quantitative polymerase chain reaction, which was performed on a StepOnePlus system (Applied Biosystems), respectively. For confirmation of *de novo* variants, we performed Trio-exome or Sanger sequencing using proband and parental samples, and confirmed the biological parentage by analyzing 10 microsatellite markers. As an exception, we included a candidate pathogenic intronic *L1CAM* variant found in a proband with consistent phenotype and inheritance, although its RNA analysis has not yet been performed.

## Results

**Fig. 1:**
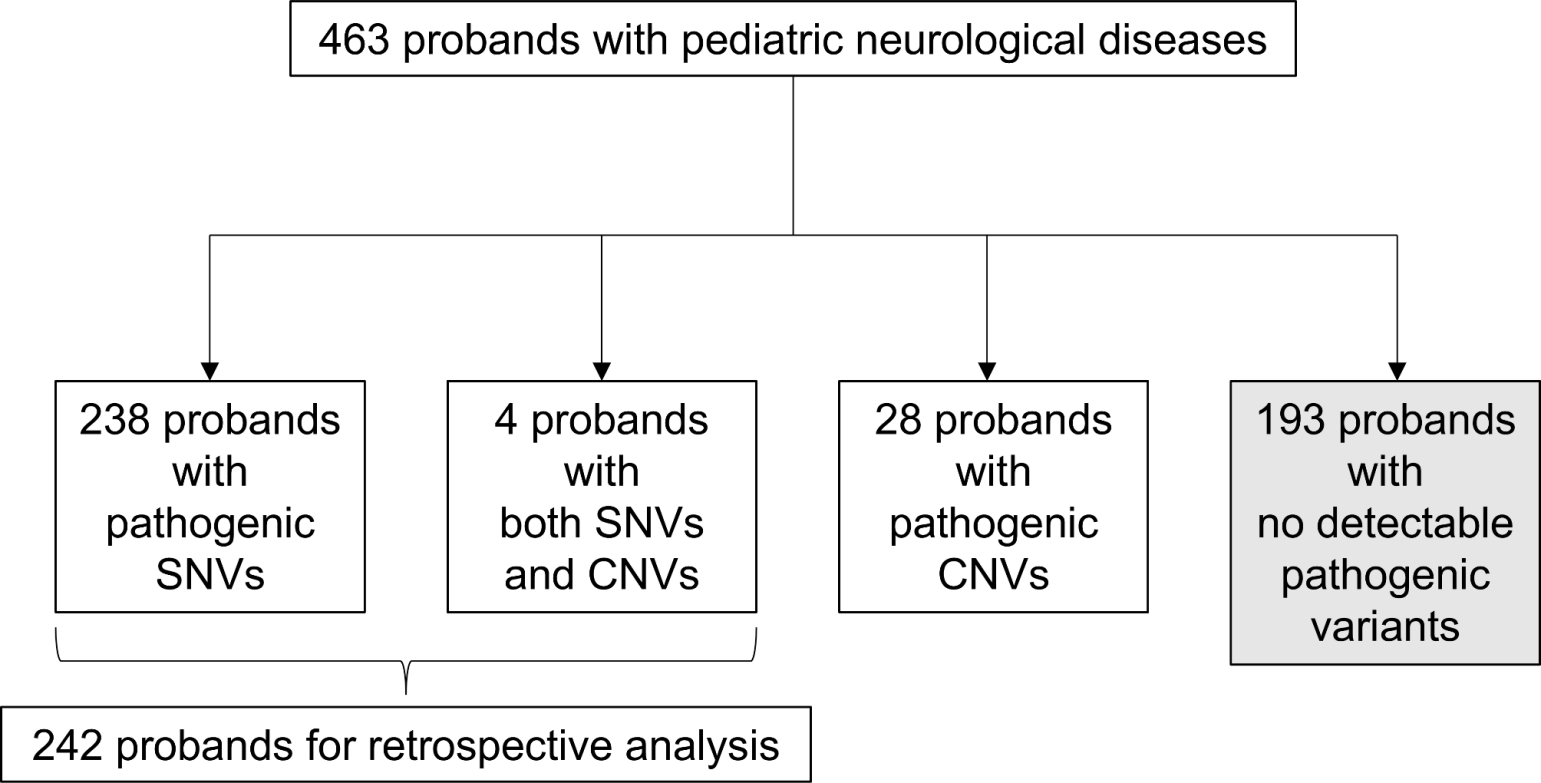
Overview of exome sequencing results for 463 probands with pediatric neurological diseases. Exome sequencing was performed for 463 probands, and pathogenic variants including copy number variants (CNVs) were found in 270 probands. Reanalysis was performed for 242 probands possessing pathogenic single nucleotide variants (SNVs)/small indels.

**Fig. 2:**
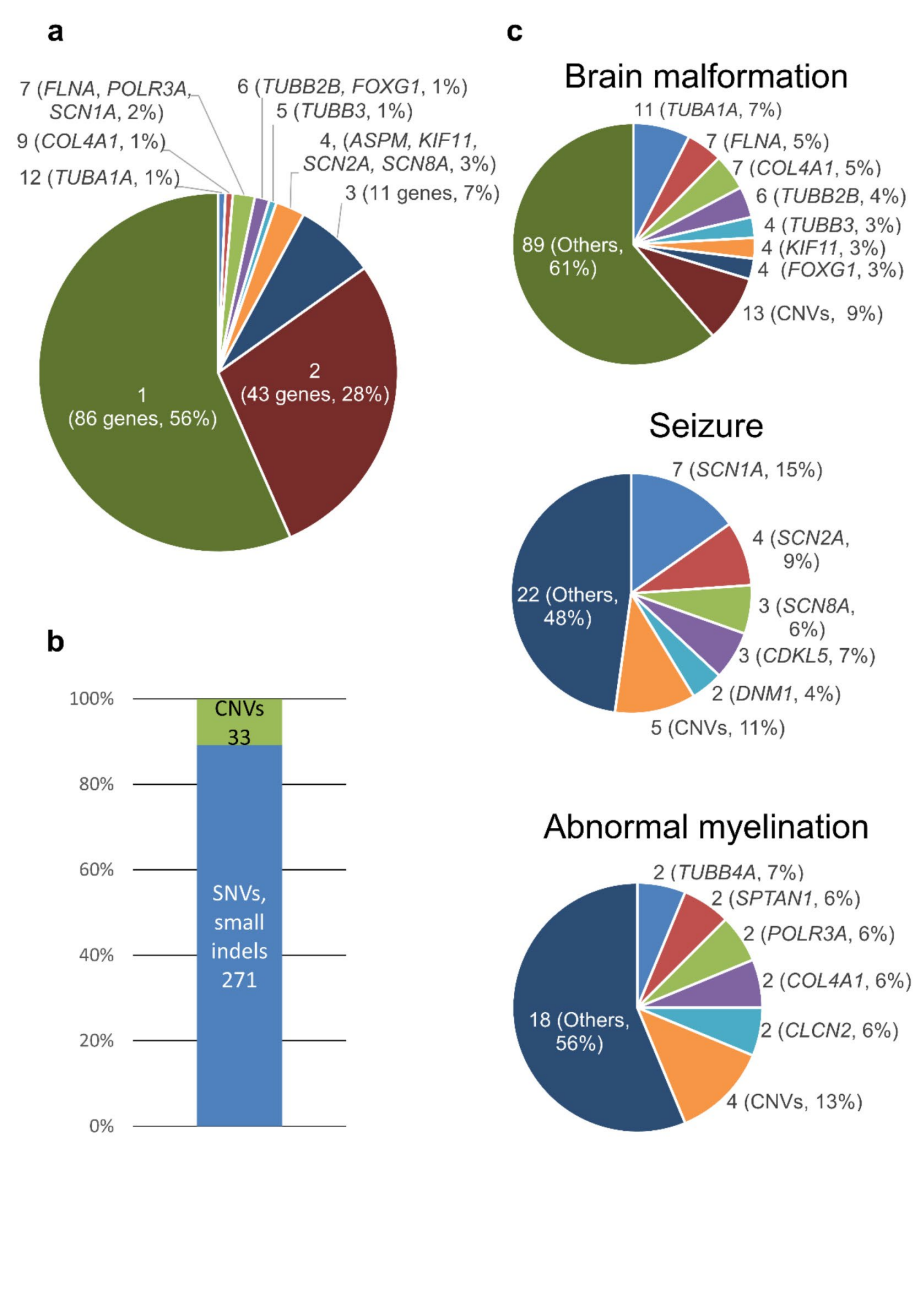
Variant types and genes in this study. (**a**) Distribution of the variant number per disease-causing genes. The number in parentheses indicates the number of genes. The genes with ≥ 4 detected variants are listed. (**b**) Number of pathogenic single nucleotide variants (SNVs), indels, and copy number variants (CNVs). (**c**) Proportion of causative genes and CNVs for three major phenotypes. The numbers indicate the number of variants.

The median depth of coverage for the 463 exomes was 78.49 (range: 34.26–309.46). Among them, pathogenic variants were detected in 270 probands (58.3%, Fig. 1). We could detect pathogenic variants—238 probands possessed SNVs and small indels, 28 probands possessed CNVs, and 4 probands possessed both SNVs and CNVs. The information for all the identified pathogenic variants is presented in Supplementary Tables S1 and S2. The most common phenotype was brain malformation (*n* = 144, 54.8%, Table 1). In 10 probands, dual phenotypes caused by multiple pathogenic variants were identified. The majority of disease inheritance was autosomal dominant (*n* = 167, 59.6%). A total of 271 SNVs and small indels were detected as pathogenic variants (Fig. 2a). *TUBA1A* variants were the most frequent among these. Additionally, 33 CNVs were found (10.9%, Fig. 2b). CNVs were observed in 9% of the probands with brain malformation, in 11% of the probands with seizure, in 13% of the probands with abnormal myelination, in 15% of the probands with neurodevelopmental delay, and in 43% of the probands with ataxia. However, no CNVs were detected in cases with involuntary movement, neuromuscular disease, or spastic paraplegia. Most pathogenic CNV regions contained genes or regions with a haploinsufficiency or triplosensitivity score of 3 (Supplementary Table S2). In the probands with brain malformation, which was most common phenotype in our cohort, *TUBA1A* was the most frequently observed gene, identified in 12 probands, including one possessing both the *TUBA1A* and *SCN8A* pathogenic variants. For seizure, *SCN1A*, which was found in 7 cases, was the most common. *TUBB4A*, *SPTAN1*, *POLR3A*, *COL4A1*, and *CLCN2* variants were each observed in two probands with abnormal myelination (Fig. 2c).

To assess the utility of gnomADv4.0 or 54KJPN in identifying *de novo* variants in probands, we evaluated the allele frequency of these variants in the databases. A total of 162 *de novo* variants in autosomal dominant or X-linked dominant genes were confirmed in 164 probands, with one proband having two *de novo* variants and one proband having three. Five recurrent *de novo* variants were also observed. Among 162 *de novo* variants, 13 variants (8.0%) were found in the databases in 14 probands, with an identical variant in two unrelated probands (Table 2). Specifically, two variants were registered in 54KJPN, 11 in gnomADv4.0 exome, and one in both 54KJPN and gnomADv4.0 exome, all with an allele frequency less than 0.001%. These data indicate that pathogenic *de novo* variants could be observed, albeit very rarely, in the large public cohort databases.

**Table 2:** De novo variants registered in 54KJPN and gnomADv4.

ID	HPO term	M-PMS	chr	Position	ref	alt	Gene, variant	54KJPN (AC/AN)	gnomADv4.0_exome (AC/AN)	gnomADv4.1 (AC/AN)	ClinVar (# of submissions)
18119	HP:0011097;HP:0001270;HP:0001249;HP:0002510	1.689	2	165,388,719	G	A	*SCN2A* NM_001040142.2:c.4913G > A,p.(R1638H)	–	1/833,110	1/1,613,944	LP (1)
19040	HP:0001274	1.860	13	26,337,623	C	T	*CDK8* NM_001260.3:c.185 C > T,p.(S62L)	–	4/1,288,948	4/1,440,984	P (3), NP (1)
19068	HP:0001250;HP:0001087;HP:0000819;HP:0008527	0.597	17	50,194,130	–	G	*COL1A1* NM_000088.4:c.1667dup, p.(G557Wfs*30)	–	1/1,458,070	1/1,607,194	P (1)
19088	HP:0000657	2.724	16	89,935,521	C	T	*TUBB3* NM_006086.4:c.1070 C > T,p.(P357L)	1/108,598	–	–	P (1)
19142	HP:0200134;HP:0001263;HP:0001249	1.611	12	51,807,101	G	A	*SCN8A* NM_001330260.2:c.5615G > A,p.(R1872Q)	–	1/628,596	1/1,613,978	P (4), NP (1)
20019	HP:0002486	4.006	17	63,964,587	C	T	*SCN4A* NM_000334.4:c.1333G > A,p.(V445M)	–	2/1,461,388	2/1,613,752	P (9)
20022	HP:0011097;HP:0001270;HP:0001249	0.857	17	31,233,095	C	T	*NF1* NM_001042492.3:c.3590 C > T,p.(A1197V)	1/108,604	–	–	Conflicting LP (1), VUS (1)
20028	HP:0001305;HP:00001249;HP:0000508;HP:0002438;HP:0002500;HP:0002079	2.004	16	89,923,432	C	A	*TUBB3* NM_006086.4:c.31 C > A,p.(Q11K)	–	1/529,184	1/1,514,138	–
20184	HP:0000252	1.288	14	28,767,529	C	–	*FOXG1* NM_005249.5:c.250del, p.(Q86Rfs*106)	–	9/913,394	9/1,060,200	P (8)
21044	HP:0002119;HP:0001250	1.543	12	49,186,784	T	C	*TUBA1A* NM_006009.4:c.53 A > G,p.(N18S)	–	1/833,110	1/1,614,176	Conflicting P (2), LP (1), VUS (2)
21210	HP:0002119;HP:0002079;HP:0002500;HP:0012502;HP:0001250;HP:0012758	2.392	12	49,186,784	T	C	*TUBA1A* NM_006009.4:c.53 A > G,p.(N18S)	–	1/833,110	1/1,614,176	Conflicting P (2), LP (1), VUS (2)
23052	HP:0032388;HP:0001321;HP:0025100	0.927	17	80,471,784	G	A	*NPTX1* NM_002522.4:c.1025 C > T,p.(A342V)	1/108,604	2/1,460,846	2/1,613,202	–
23176	HP:0001355;HP:0000369	0.477	7	105,112,579	–	T	*KMT2E* NM_182931.3:c.4824dup, p.(L1610Ffs*259)	–	1/628,774	1/1,613,826	P (1), LP (1)
23245	HP:0001263;HP:0000729;HP:0007018;HP:0000252;HP:0000588	1.636	16	70,663,910	G	A	*MTSS2* NM_138383.3:c.2011 C > T,p.(R671W)	–	2/1,397,422	2/1,549,794	P (1), LP (3)

M-PMS, Maximum PhenoMatch score; AC, Allele count; AN, Allele number; P, Pathogenic; LP, Likely pathogenic; VUS, Uncertain significance; NP, Not provided.

**Fig. 3:**
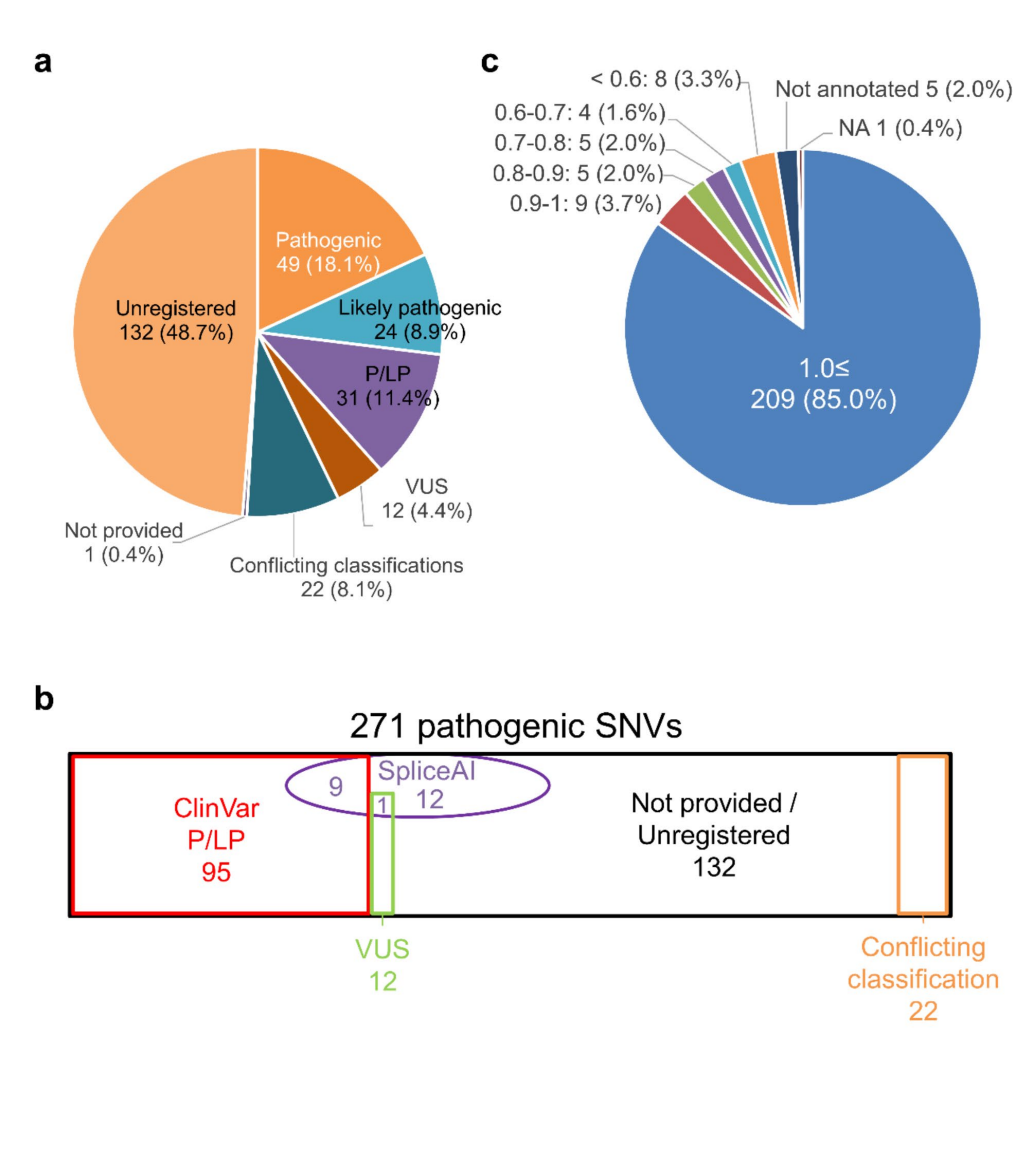
Impact on pathogenic variants by each annotation. (**a**) Registration status of the pathogenic variants in ClinVar. Classifications are shown with variant numbers and percentage. P/LP, pathogenic/likely pathogenic; VUS, variant of uncertain significance. (b) Relationship between ClinVar and SpliceAI annotations in 271 pathogenic single nucleotide variants (SNVs). The numbers indicate the number of variants. (**c**) Distribution of max score in the PhenoMatcher module for each gene. NA, Not available phenotype data.

Next, we evaluated the utility of annotation based on ClinVar pathogenicity classifications. Among the SNVs and small indels identified in this study, 38.4% were registered in ClinVar with P or LP classification (Fig. 3a), which underscores the immense utility of this database. Variants unregistered in ClinVar accounted for 48.7% of the variants.

Among 24 intronic variants, SpliceAI could predict aberrant splicing with delta score equal or above 0.2 in 22 variants (91.7%). Among 22 variants, only nine variants were registered as P or LP in ClinVar (Fig. 3b). Notably, we found four variants that were located more than 10 bp away from the exon–intron boundary and predicted aberrant splicing using SpliceAI (Table 3, and Supplementary Figure S2). Among these variants, the splicing change in *WDR37*, *CEP290* has been confirmed in previous studies^9,25^. Three of four variants have been registered as P or LP in ClinVar, including a *WDR37* variant, which was registered by us^9^.

**Table 3:** Intronic variant which is affected splicing within 11–50 bp from exon.

ID	HPO term	M-PMS	chr	Position	ref	alt	Inheritance	Gene, variant	SpliceAI delta score AG|AL|DG|DL	ClinVar (# of submissions)
17132	HP:0000113;HP:0002023;HP:0001249;HP:0001320;HP:0002282	0.714	10	1,103,570	TCTT	–	*de novo*	*WDR37* NM_014023.4:c.727 − 27_727-24del, p.(Ser246_Glu247insLeuCysGlnLysLysLeuLysIleSerArgLysCysLeuPheTrpProSerLeuTrpGlnGln)	0.44|0.14|0.01|0.01	Likely_pathogenic (1)
20065	HP:0006855	2.055	12	88,068,657	A	T	Paternal	*CEP290* NM_025114.4:c.6012-12T > A	0.34|0.95|0.01|0.04	Pathogenic (2)
21038	HP:0001274;HP:0000365;HP:0002119	1.528	X	153,869,687	A	C	Maternal	*L1CAM* NM_001278116.2:c.1124-24T > G	0.99|0.18|0.00|0.00	
21118	HP:0002119;HP:0001172	2.019	X	153,863,391	C	T	*de novo*	*L1CAM* NM_001278116.2:c.3531-12G > A	0.97|0.65|0.04|0.00	Pathogenic (2)

M-PMS, Maximum PhenoMatch score; AG, Acceptor gain; AL, Acceptor loss; DG, Donor gain; DL, Donor loss.

We also evaluated the utility of a phenotype annotation tool, the PhenoMatcher module (https://github.com/liu-lab/exome_reanalysis). Approximately 95% of the candidate genes had maximum PhenoMatch scores of 0.6 or above, and 85.1% of the candidate genes had scores of 1.0 or above (Fig. 3c). Because the maximum PhenoMatch score of 0.3 was used as a threshold in a previous study^23^, these data suggest a good correlation between genes and phenotypes, and demonstrate the utility of prioritizing candidate genes.

In this analysis, we combined the gVCF files of probands using GLNexus. In this process, a *FOXG1* variant was filtered out (Supplementary Figure S3), which was called in the gVCF. Multisample calling is recommended in GATK best-practice; however, it should be borne in mind that true but low-quality calls might be excluded in the quality filtering step.

## Discussion

In this study, we found pathogenic SNVs, small indels, and CNVs in 270 of 463 probands with rare pediatric neurological diseases. Among the identified pathogenic variants, CNVs were observed in approximately 10% of the probands (Fig. 1). Intragenic CNVs were reported to account for 9.8% of the pathogenic or likely pathogenic variants identified through a panel analysis of Mendelian disease genes in a previous study^26^. In neurological disease cohorts, CNVs detected based on exome sequencing data accounted for 3.8%, 2%, and 1.2% of the variants in neuropathies, movement disorders, and muscle diseases, respectively^27^. In our cohort, the CNV detection rate for ataxia was 43%, which is higher compared with the 1% CNV rate reported among the 36 known genes associated with cerebellar ataxia^28^. This discrepancy may be attributed to differences in cohort characteristics, disease classification criteria, and the small samples size in the present study; however, it is noteworthy that CNVs contribute to the improved diagnostic rate of ataxia. These results confirm that exome sequencing, including CNV analysis, is useful in the genetic diagnosis of pediatric neurological diseases^1,29^.

We retrospectively evaluated the impact of four annotations for identifying pathogenic variants in probands with pediatric neurological diseases. To date, approximately 3 million pathogenic variants have been registered in the ClinVar database. However, 132 out of the 271 pathogenic variants in our cohort were not registered in this database. On the contrary, we also found that ClinVar annotation is of immense value, as 38.1% of the candidate variants had been registered in the ClinVar database as pathogenic or likely pathogenic. These variants could be easily identified by checking the ClinVar annotation, which reduces the burden of manual analysis. Because the ClinVar database is rapidly growing, utilizing the latest information may increase diagnostic yield. For example, the *HSD17B4* c.350 A > T variant (ID: 18081) was not registered in ClinVar at the time of publication of the previous report^17^, but has been registered as “pathogenic” in the latest ClinVar. Because the VCF file format information in ClinVar is updated monthly, the ClinVar annotations should be regularly updated during (re-)analysis.

Notably, four intronic variants have been identified as P/LP or as a strong candidate. These variants were located between positions 11 and 50 bp away from the exon–intron boundary. SpliceAI is highly sensitive in predicting cryptic new donor or acceptor sites and the loss of canonical splice sites^30^. Delta scores for either splice site gain or loss were 0.95 or above in three variants, and 0.44 in one variant (Table 2), where three of the four variants being registered as P or LP in ClinVar, highlighting the usefulness of combining ClinVar and SpliceAI annotations for intronic variants. Notably, a *L1CAM* variant (NM_001278116.2:c.1124-24T > G) was not registered in ClinVar; thus, the SpliceAI annotation could exclusively contribute to the possible genetic diagnosis of this proband, although RNA analysis should be performed. Depending on the capture efficiency, expanding analysis region of introns beyond 50 bp from the exon–intron boundary may increase the detection of pathogenic variants in undiagnosed cases. However, our analysis showed that the number of pathogenic intronic variants decreased from 20 within 10 bp to four in the 11–50 bp range, suggesting that the further a variant is from the canonical splice site, the less likely it is to impact splicing. Additionally, as the analysis range of introns expands, the accuracy of called variants decreases^3^, and analysis time and cost may increase. Considering these factors, our findings suggest that extending the analysis range to 50 bp is practically useful for detecting pathogenic intronic variants in the routine pipeline of exome sequencing in combination with ClinVar and SpliceAI annotations.

We found that 13 *de novo* variants in 14 probands, with very low allele frequencies, were registered in large public cohort databases. Nine variants were registered as pathogenic or likely pathogenic in ClinVar, but two were classified as having conflicting classifications of pathogenicity and two were unregistered. Pathogenic heterozygous variants for dominant severe pediatric diseases might still be observed due to factors, such as incomplete penetrance, imprinting, or mosaicism^1^. *TUBB3* variants cause fibrosis in extraocular muscles and cortical dysplasia, which have complete penetrance with a broad spectrum of phenotypes, including mild developmental delay^31^. Therefore, we believe that the broad disease phenotypes of *TUBB3*-related disorders may lead to the identification of one individual harboring the *TUBB3* (c.1070 C > T) variant in 54KJPN. On the contrary, somatic mosaicism may be involved in the case of *FOXG1* variants. The c.250del *FOXG1* variant was registered as pathogenic with three stars in ClinVar, but was found in nine individuals in gnomADv4.1. However, the allele balance of eight variant carriers was in the 0.2–0.25 range, and one variant carrier was in the 0.25–0.3 range. Although our case also shows an allele balance of 0.33, these findings suggest that c.250del could occur as a somatic variant. Therefore, we should be mindful of the fact that very rare variants in large cohort data can be pathogenic *de novo* variants.

The numbers of genes responsible for Mendelian disorders is continuously increasing. Therefore, updating annotations concerning the gene–disease–phenotype associations will be essential to identify pathogenic variants in recently reported genes in exome (re)analysis^32^. In this study, we utilized the PhenoMacher module for prioritizing candidate genes. This program allows for the dynamic incorporation of new knowledge regarding the gene–disease–phenotype associations by updating the most informative common ancestor matrix, which can be created with three datasets (hp.obo, phenotype.hpoa, and genes_to_phenotype.txt; available from the human phenotypic ontology webpage). Therefore, by updating matrix using the three updated datasets, the risk of overlooking recently reported genes can be minimized. Although the effectiveness of PhenoMatcher in identifying the causative genes in pediatric neurological diseases has not been reported, our cohort, with 95% of the probands having a score of 0.6 or higher, could provide valuable information for determining the cutoff in pediatric neurological diseases. In practice, combining these annotations with predictions of the effects of genetic variants, such as BayesDel^33^, CADD^34^, PolyPhen-2^35^, or REVEL^36^, may facilitate the identification of pathogenicity, especially for variants not annotated in ClinVar^37^.

The limitations of this study include the small sample size, which does not encompass the entire spectrum of pediatric neurological diseases, and the potential for selection bias considering the cohort consists only of probands collected in our laboratory. Additionally, only a limited number of annotation tools were utilized.

In summary, evaluation of the utility of the various annotation tools in identifying pathogenic variants suggests that combination of multiple annotations, such as ClinVar and SpliceAI score, can improve the diagnostic yield of rare diseases. Careful examination is required to avoid overlooking intronic and very rare *de novo* variants in the general populations.

## Electronic supplementary material

Below is the link to the electronic supplementary material.

Supplementary Material 1

Supplementary Material 2

## Data Availability

All data obtained in this study are available from the corresponding author (H.S.) upon reasonable request.
