# Supplementary material for: Identifying pathogenic variants in rare pediatric neurological diseases using exome sequencing
Source: Sci Rep. 2024 Oct 21;14:24746. doi: 10.1038/s41598-024-75020-0 (PMC11494122; doi:10.1038/s41598-024-75020-0)
Supplement: Supplementary file 1 — Supplementary Material 1 [file 41598_2024_75020_MOESM1_ESM.pdf]

Identifying pathogenic variants in rare pediatric neurological diseases using exome sequencing

Kazuyuki Komatsu, Mitsuhiro Kato, Kazuo Kubota, Shinobu Fukumura, Keitaro Yamada, Ikumi Hori, Kenji Shimizu, Sachiko Miyamoto, Kaori Yamoto, Takuya Hiraide, Kazuki Watanabe, Shintaro Aoki, Shogo Furukawa, Taiju Hayashi, Masaharu Isogai, Takuma Harasaki, Mitsuko Nakashima and Hirotomo Saitsu

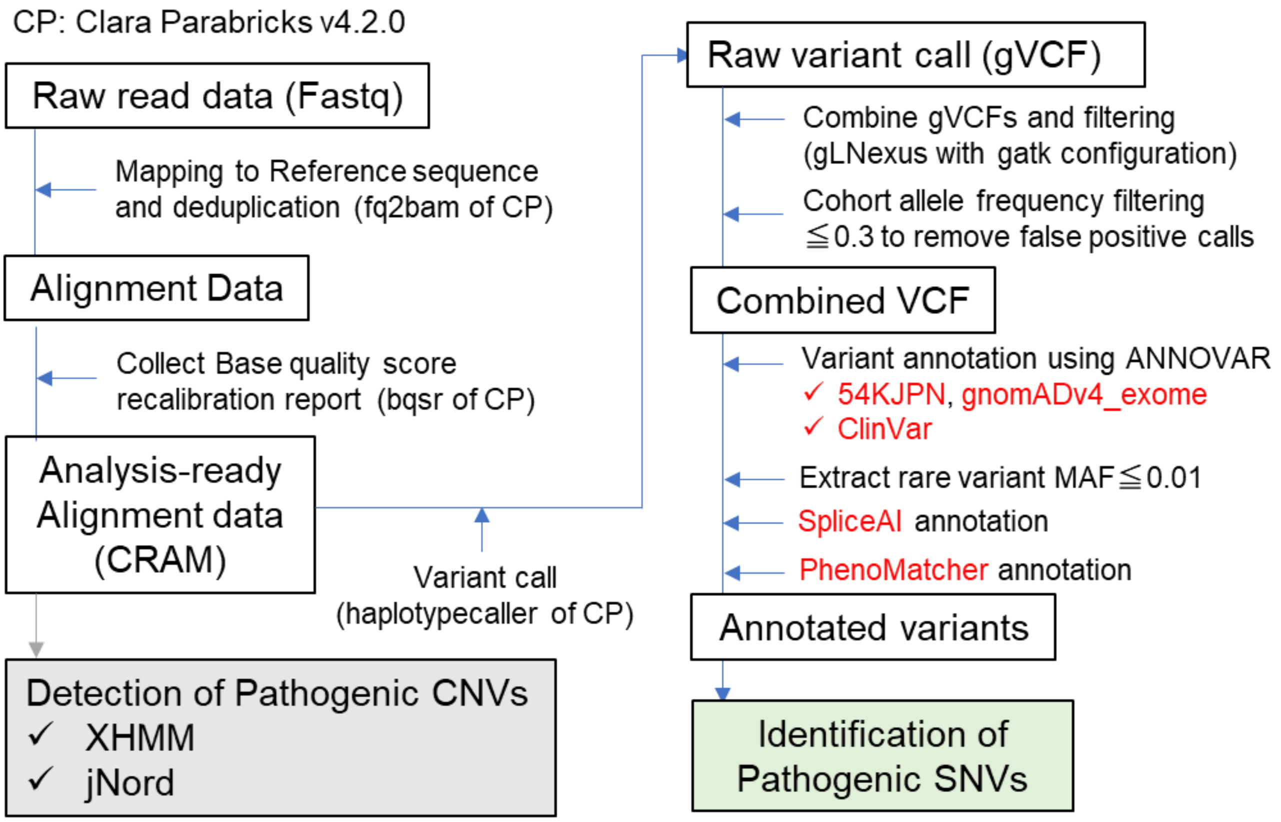

Supplementary Figure S1. Our workflow of exome data (re)analysis

CRAM, compressed reference-oriented alignment map; CNVs, copy number variants; SNVs, single nucleotide variants; VCF, Variant Call Format; MAF, minor allele frequency

XHMM, eXome-Hidden Markov Model[1].

jNord[2] is available at GitHub (https://github.com/bitsyamagu/jnord).

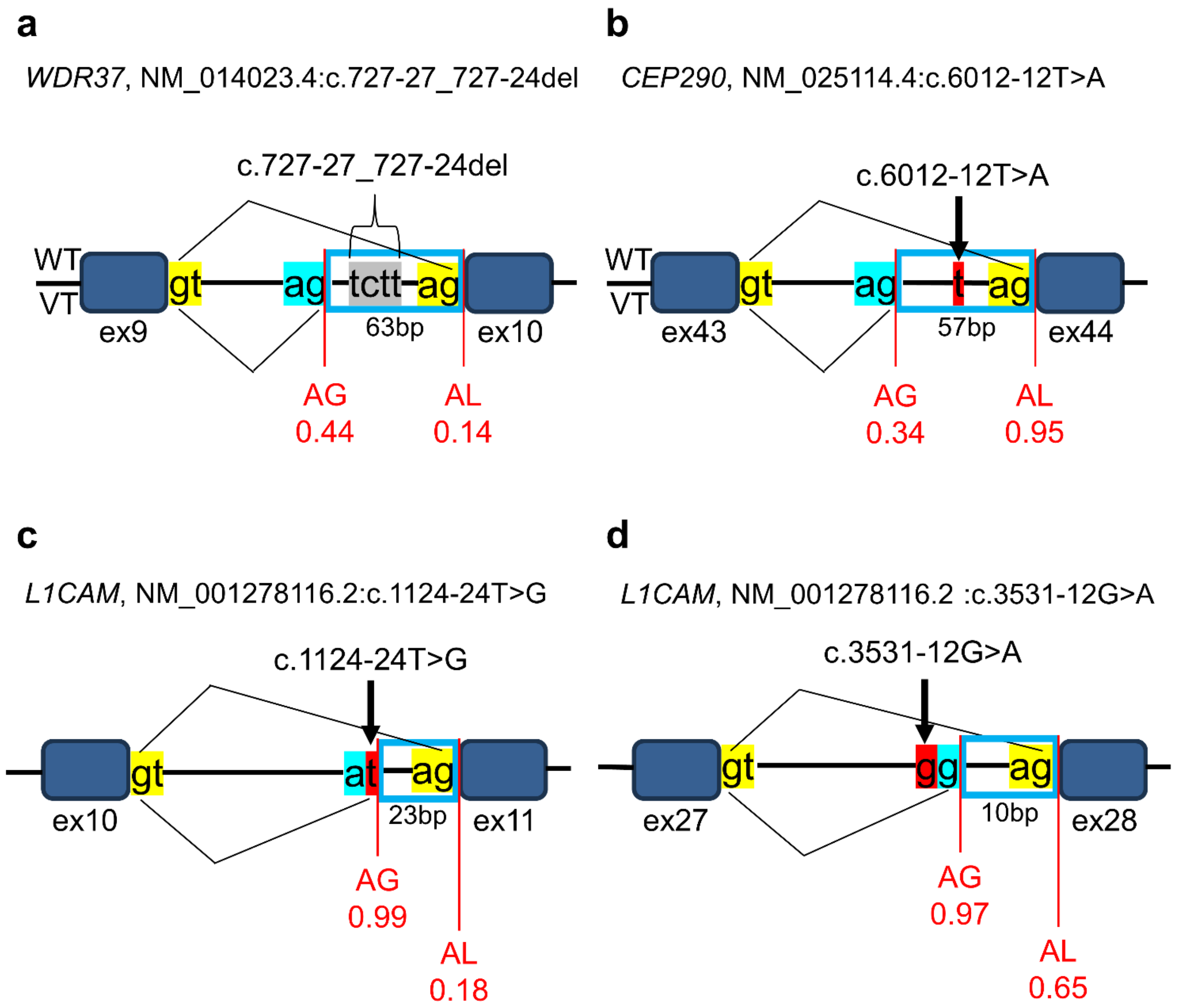

Supplementary Figure S2. SpliceAI prediction of aberrant splicing by four intronic variants 10-bp away from the exon-intron boundary

SpliceAI prediction of aberrant splicing in *WDR37* (ID:17132, **a**), *CEP290* (ID:20065, **b**), and *L1CAM* (ID:21038, **c**; ID:21118, **d**) variants. Canonical splice sites are highlighted in yellow, while newly predicted splice sites by SpliceAI are highlighted in sky blue. Variants predicted to cause aberrant splicing are marked in red. Delta scores of each splice sites are shown with red lines. The proband of *WDR37* (**a**) was already reported[3] and aberrant splicing caused by the *CEP290* variant (**b**) has been previously confirmed[4]. AG, Acceptor gain; AL, Acceptor loss; DG, Donor gain; DL, Donor loss.

a

No.	chr	Genome position	ref	alt	gene	variant	read GT:AD:DP
20184	14	28767529	C	-	*FOXG1*	NM_005249.5:c.250del,p.(Q86Rfs*106)	0/1:6,3:9

b

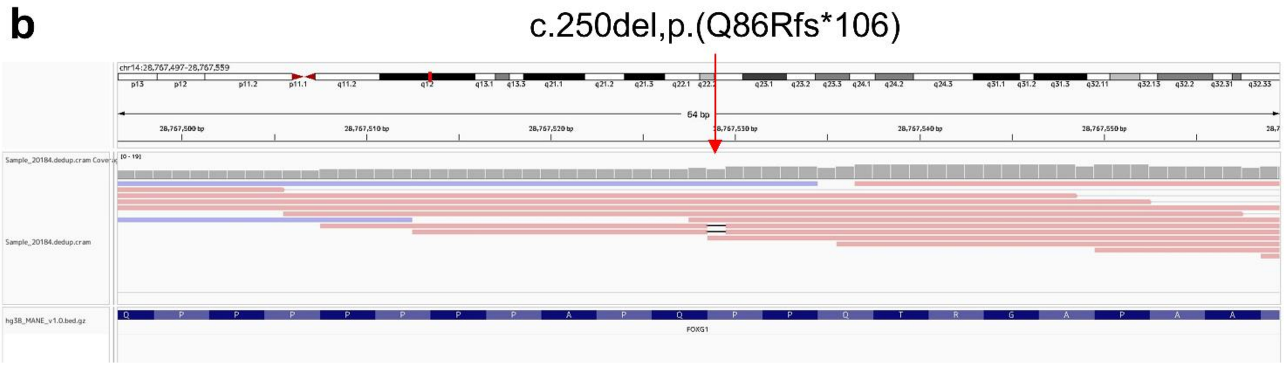

c

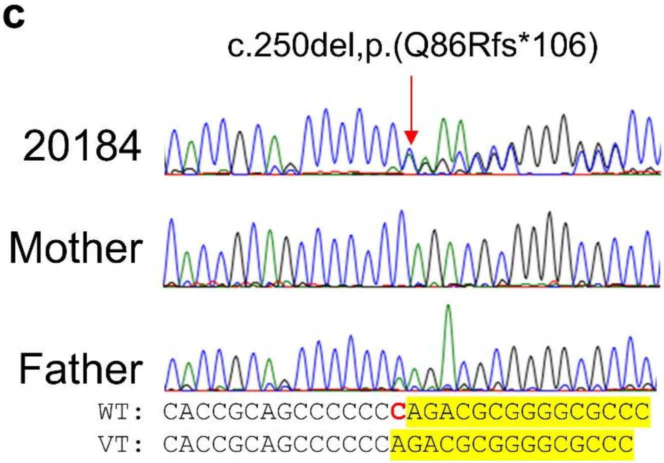

Supplementary Figure S3. Confirmation of *FOXG1* variant. (**a**) Read information from the VCF file before combining gVCF files. GT, Genotype; AD, Allelic depth (reference, alteration), DP Approximate read depth. (**b**) IGV view of the variant, showing low depth of coverage. (**c**) Sanger sequencing confirmed the *de novo FOXG1* variant.

Reference

1 Fromer, M. *et al.* Discovery and statistical genotyping of copy-number variation from whole-exome sequencing depth. *Am J Hum Genet* **91**, 597-607, doi:10.1016/j.ajhg.2012.08.005 (2012).

2 Uchiyama, Y. *et al.* Efficient detection of copy-number variations using exome data: Batch- and sex-based analyses. *Human mutation* **42**, 50-65, doi:10.1002/humu.24129 (2021).

3 Samejima, M., Nakashima, M., Shibasaki, J., Saitsu, H. & Kato, M. Splicing variant of *WDR37* in a case of Neurooculocardiogenitourinary syndrome. *Brain Dev* **46**, 154-159, doi:10.1016/j.braindev.2023.11.007 (2024).

4 Tsurusaki, Y. *et al.* The diagnostic utility of exome sequencing in Joubert syndrome and related disorders. *J Hum Genet* **58**, 113-115, doi:10.1038/jhg.2012.117 (2013).
